# Can video mobile phones improve CPR quality when used for dispatcher assistance during simulated cardiac arrest?

**DOI:** 10.1111/j.1399-6576.2008.01779.x

**Published:** 2009-01

**Authors:** S R BOLLE, J SCHOLL, M GILBERT

**Affiliations:** 1 Norwegian Centre for Telemedicine, University Hospital of North NorwayTromsΦ, Norway; 2 Department of Emergency Medicine, University Hospital of North NorwayTromsΦ, Norway

## Abstract

**Background:**

Because mobile telephones may support video calls, emergency medical dispatchers may now connect visually with bystanders during pre-hospital cardio-pulmonary resuscitation (CPR). We studied the quality of simulated dispatcher-assisted CPR when guidance was delivered to rescuers by video calls or audio calls from mobile phones.

**Methods:**

One hundred and eighty high school students were randomly assigned in groups of three to communicate via video calls or audio calls with experienced nurse dispatchers at a Hospital Emergency Medical Dispatch Center. CPR was performed on a recording resuscitation manikin during simulated cardiac arrest. Quality of CPR and time factors were compared depending on the type of communication used.

**Results:**

The median CPR time without chest compression (‘hands-off time’) was shorter in the video-call group vs. the audio-call group (303 vs. 331 s; *P*=0.048), but the median time to first compression was not shorter (104 vs. 102 s; *P*=0.29). The median time to first ventilation was insignificantly shorter in the video-call group (176 vs. 205 s; *P*=0.16). This group also had a slightly higher proportion of ventiliations without error (0.11 vs. 0.06; *P*=0.30).

**Conclusion:**

Video communication is unlikely to improve telephone CPR (t-CPR) significantly without proper training of dispatchers and when using dispatch protocols written for audio-only calls. Improved dispatch procedures and training for handling video calls require further investigation.

Video calls are now widely available to the public on 3G mobile phones. Dispatchers in centers with technology allowing reception of video calls from the public may have more information from the scene of accidents and other medical emergencies. If dispatchers are enabled to see patients, bystanders and rescue attempts, the information may help dispatchers plan and coordinate resources, and possibly improve instructions given to the caller. Little is known about how this technology would influence outcome.

Standard protocols for telephone CPR-instructions (t-CPR) have been developed and tested, and national and international protocols for t-CPR are being used currently.^1^–^3^ Previous studies have yielded conflicting results on the efficacy of t-CPR, and scripted telephone instructions may need change.^1^,^4^–^6^

We hypothesized that video communication could improve the quality of lay people CPR by enhancing communication between bystanders and dispatchers during t-CPR. In a previous study, we found that video communication can improve dispatchers’ understanding of the rescuer's situation and improve the guidance they provide.^7^ We now report the effects of video calls on t-CPR quality during the same trials. We conducted a randomized-controlled study comparing lay bystanders’ CPR performance using video calls or audio calls in a standard cardiac arrest simulation on a recording CPR manikin.

## Methods

### Study design and population

Students from three different high schools in Tromsø, Norway, volunteered as lay bystanders. We used high-school students, because they adapt more quickly to new mobile phone technology and represent the future users. We also wanted to avoid that the mere use of communication technology would disturb resuscitation attempts. We, therefore, divided the students into groups of three to allow two persons for CPR while the third person took care of communication with the dispatch center.

The study population was selected during regular school hours without prior warning. All students gave informed consent. Twenty groups participated each day, totaling 180 students in 60 groups. Six experienced nurse dispatchers were recruited from the University Hospital Emergency Medical Dispatch Centre (AMK-Tromsø). Two dispatchers participated each day, performing 10 sessions of t-CPR each during the study. We randomly selected the first dispatcher and the first communication mode tested each day. The dispatcher and communication mode were switched for each session. Trials were performed during December 2006 and January 2007. The study was accepted by the Norwegian Social Science Data Services.

### Communication technology

Rescuers communicated either via mobile phone audio calls on a Sony Ericsson K800i (Sony Ericsson Mobile Communications AB, Lund, Sweden) or via combined audio and video calls on a Nokia N90 mobile telephone (Nokia Corporation, Helsinki, Finland). To improve the ecological validity of the study, the default audio setting was used for each mode. The loudspeaker function was activated by default when instructions were delivered via the Nokia N90 video phone. When t-CPR instructions were delivered via the audio-only mobile phone (K800i), the loudspeaker function was not used. We thus studied ‘loudspeaker video’-assisted instructions compared with non-loudspeaker audio-call instructions.

When delivering audio-call instructions, dispatchers used a telephone with a standard headset. For video-call instructions, dispatchers used a laptop with a UMTS (3G) card, video camera, video communication software (VT-phone, Dilithium Inc., Petaluma, California) and a standard headset. The mobile network service provided video calls with the 3G-324M protocol, with a maximum speed of 64 kbit/s during the study period.

During video calls, the caller could see the dispatcher on the phone's small color screen. The phone's camera could be pointed by the caller, and this image was seen by the dispatcher, who could also ask the caller to reposition the phone camera. In most cases, the dispatcher would want to see the manikin and actions of resuscitation carried out by the two others in the group of three.

During audio calls, the caller was expected to convey all information to the other two team members as is normal procedure when several persons perform t-CPR.

### Methods and measurements

Dispatchers used the national Norwegian criteria-based dispatch protocol updated to recent CPR protocols,^3^,^8^ and were told to expect calls from any type of simulated pre-hospital medical emergency. None of the dispatchers was trained to use video communication for t-CPR. Before the first session, they were briefly instructed on how to start and stop video calls and audio calls.

We used a standard training manikin to simulate cardiac arrest (Laerdal Resusci® Anne Manikin, Laerdal, Stavanger, Norway). Each student group was given a written description of the simulated emergency (modified after Whitfield^9^):

‘You are called to help a person who has collapsed. When you enter the room you will see a training dummy supposed to be an adult about 50 years old. When this experiment starts, you should treat this ‘person’ until we tell you to stop. We will give you a mobile phone which connects you to a nurse at the hospital dispatch center. [For the video group: The telephone has a camera, and the person carrying the phone should film what you do.] You can talk with the nurse if you need help to treat the person. In the room a person will be filming, but will not answer questions. If you have questions you must use the phone. The scenario will last about 10 min. This may seem like a long time, but please continue to treat the person until we tell you to stop.’

### Data collection and analysis

CPR performance was recorded with the resuscitation manikin and a Laerdal PC Skill Reporting System®. Such data have been validated and recommended when evaluating CPR performance.^10^,^11^

Data collection started when students entered the scene, and lasted for 10 min. Demographic variables were collected with written questionnaires. Data were recorded in a database and analyzed using the R language and environment for statistical computing.^12^ Differences among groups were assessed by the Wilcoxon rank sum test. A significance level of *P*<0.05 indicated statistical significance. High-quality video recording was carried out discreetly for further study.

### Sample size

The study sample size was calculated using the power *t*-test of the *R* environment^12^ to detect a 20% improvement for compressions delivered per minute, with α=0.05 and β=0.2. A previous study of lay providers reported an average number of compressions delivered per minute of 39 (standard deviation 11).^13^ Computing this gave a required minimum sample size of 25 for each communication mode. We also calculated the required sample size based on our own data from a small pre-study, with an average number of compressions delivered per minute of 37.4 (standard deviation 9.4), giving a required minimum sample size of 21 groups. We, therefore, planned for 30 groups of students for each communication mode to account for potential drop-outs and for using non-parametric tests.

## Results

Study groups had similar demographics and previous CPR training. The mean age was 17.9 years in the audio group (range 16–32 years) and 17.3 years in the video group (range 15–34 years). 34.4% of the participants in the audio group and 26.7% in the video group were males. Seventy one percent of the students in the audio group and 73% in the video group had some kind of CPR training during the last 3 years. One male and five female dispatchers, with a mean age of 33.5 years (range 28–51 years), provided telephone CPR instructions.

Video recordings carried out during the trial show that dispatchers adapted their instructions based on the input they receive from the rescuers. During video calls, dispatchers often responded to what they saw, clarified misunderstandings or gave more detailed instructions when they saw that rescuers did not perform as they wanted. Verbal support was often provided on how to open the airway and how to perform ventilations when they saw rescuers struggling with these procedures.

During the 60 cardiac arrest simulations, five cases had neither chest compressions nor ventilations registered by the recording manikin: one during video and four during audio sessions. Video recordings showed that students in these five scenarios believed that the manikin was breathing, and in cooperation with the dispatcher nurse, placed the manikin in the recovery position without attempts of resuscitation. Among the remaining scenarios, there were another four cases where no ventilations were registered: one during video and three during audio sessions. Our video recordings for these four scenarios show that students attempted ventilations, but failed to open airways.

**Fig. 1:**
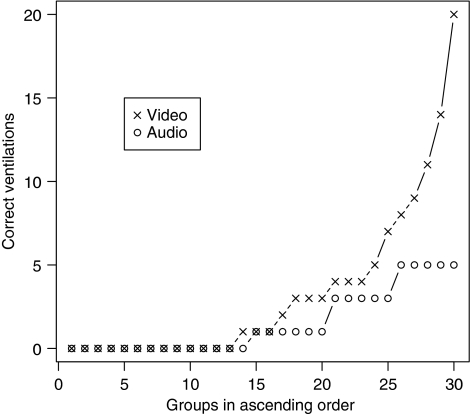
The number of correct ventilations for each communication mode. Groups are arranged in ascending order.

Table 1 shows the resuscitation performance for video and audio groups recorded by the manikin. The five scenarios without resuscitation attempts are excluded. The total time without chest compression (hands-off time) was significantly shorter for video calls (*P*=0.048). No significant differences between video and audio groups were found for the other variables of CPR quality. A contributing factor was that a large number of groups were unable to perform any of the CPR tasks correctly. The best performers of ventilations during video calls were, however, noticeably better than the best performers during audio calls (Fig. 1).

**Table 1:** CPR performance in two groups of lay rescuers guided by dispatchers via audio-only or video mobile phones.

	Audio group (*n*=26)	Video group (*n*=29)	*P*-value
*Compressions*			
Total number of compressions	460 (405–575)	480 (418–613)	0.23
Average depth (mm)	38 (30–41)	37 (31–42)	0.83*
Average rate (*n*/min)	110 (104–119)	114 (93–119)	0.75*
Average number per minute	61 (55–73)	62 (55–74)	0.34
Proportion done without error	0.08 (0.03–0.18)	0.09 (0.01–0.22)	0.50
Proportion done to correct depth (38–51 mm)	0.31 (0.16–0.55)	0.35 (0.11–0.59)	0.53
Proportion with correct hand position	0.50 (0.18–0.63)	0.45 (0.26–0.62)	0.52
Proportion done with full release	1.00 (1.00–1.00)	1.00 (1.00–1.00)	0.83
Time to first compression (s)	102 (84–142)	104 (74–123)	0.29
Total hands-off-chest time (s)	331 (300–346)	303 (283–329)	0.05
*Ventilations*			
Total number of ventilations	24 (17–33)	28 (10–32)	0.50
Average ventilation volume (ml)	1356 (953–1466)	1163 (734–1511)	0.74*
Average number per minute	4 (4–5)	4 (4–5)	0.30
Proportion without error	0.05 (0.00–0.17)	0.09 (0.00–0.27)	0.32
Proportion with correct volume (500–800 ml)	0.06 (0.01–0.21)	0.11 (0.00–0.28)	0.30
Time to first ventilation (s)	205 (138–260)	176 (119–214)	0.16

Median (interquartile range) performance in two groups of lay people delivering CPR with telephone-mediated support from a dispatch center nurse. One group used a mobile phone with a video camera (‘Video group’), the other a mobile phone with only audio communication (‘Audio group’). *P*-values were computed using the Wilcoxon single-sided

* (or two-sided) rank sum test.

## Discussion

Bystander CPR in dramatic pre-hospital situations is frequently supported by telephone-mediated advice from medical dispatch centers. Misunderstandings, language barriers and severe stress may distort communication between the two parties when ‘blind’ communication by phone is their only link. Dispatchers reported video communication as an improvement in such settings.^7^ We could not prove that the objective quality of key variables such as compressions or ventilations improved when video communication was added to t-CPR (Table 1).

However, the video groups had a significantly shorter hands-off time. This is in line with suggestions by some of the dispatchers.^7^ Less hands-off time is considered a significant contributor to survival during pre-hospital CPR for cardiac arrest.^14^–^16^ This finding may not be clinically important because time savings were <10% and the first compression was not started earlier. It is interesting that the introduction of new technology did not waste time, even though neither the dispatchers nor the rescuers had previous experience with video calls. An important time-saving factor could be the loudspeaker function on the mobile phones during video calls, enabling the dispatcher to instruct the whole CPR team simultaneously.

During audio calls, more groups failed to identify respiratory arrest or failed to open the airway. We believe this was caused by dispatchers using the images for more targeted instructions for airway opening and ventilations. The same observations may explain why our data indicate that video calls may improve the quality of ventilations for some rescuers (Fig. 1).

Dispatchers had no training in the use of video communication in this setting, and had to discover the potentials and limitations of the technology themselves. We also used a criteria-based dispatch protocol designed for audio-only t-CPR. This might have limited the positive findings during video communication.

Proper use of health information systems often requires a socio-technical approach that deals with technical and organizational issues.^17^,^18^ We believe proper training of dispatchers and revised dispatch protocols for t-CPR by video calls may improve outcome compared with audio-only calls. Further studies are needed to elucidate these potentials.

The mobile video telephones and the network bandwidth used during this study did not allow very high-quality picture, and dispatchers noted that pictures were often inadequate to identify the details of CPR performance.^7^ Future technology improvements will probably change this and provide other benefits from video calls.

Most witnessed cardiac arrests occur in the home where the presence of several potential rescuers is unlikely,^19^,^20^ and use of video phones for one-rescuer t-CPR should be studied.

We studied high school students, a population more likely to adapt to the use of new mobile technology, but not a risk population for cardiac arrest. However, only those who know how to use video phones will be likely users during medical emergencies. In the future, when more people are accustomed to video conferencing through mobile phones, this limitation would not apply.

## Conclusion

This study does not support implementing routine use of video calls from mobile phones when the public communicates with dispatch centers during CPR. Our study was limited by the low-quality video received from phones, and lack of prior training of dispatch personel in using video calls. Further studies may demonstrate improved clinical outcome for video calls, given such improvements. We conclude that current video communication is unlikely to improve t-CPR significantly without proper training of dispatchers and with dispatch protocols written for audio-only calls.
